# Homozygous Inactivating Mutation in *NANOS3* in Two Sisters with Primary Ovarian Insufficiency

**DOI:** 10.1155/2014/787465

**Published:** 2014-06-26

**Authors:** Mariza G. Santos, Aline Z. Machado, Conceição N. Martins, Sorahia Domenice, Elaine M. F. Costa, Mirian Y. Nishi, Bruno Ferraz-de-Souza, Soraia A. C. Jorge, Carlos A. Pereira, Fernanda C. Soardi, Maricilda P. de Mello, Andrea T. Maciel-Guerra, Gil Guerra-Junior, Berenice B. Mendonca

**Affiliations:** ^1^Unidade de Endocrinologia do Desenvolvimento, Laboratório de Hormônios e Genética Molecular/LIM-42, Hospital das Clínicas da Faculdade de Medicina da Universidade de São Paulo, Avenida Dr. Eneas de C Aguiar 155, 2 andar Bloco 6, 05403-900 São Paulo, SP, Brazil; ^2^Laboratório de Carboidratos e Radioimunoensaios/LIM-18, Hospital das Clínicas da Faculdade de Medicina da Universidade de São Paulo, Avenida Dr. Arnaldo 455, 01246-903 São Paulo, SP, Brazil; ^3^Laboratorio de Imunologia Viral, Instituto Butantan, Avenida Vital Brasil 1500, 05503-900 São Paulo, SP, Brazil; ^4^Centro de Biologia Molecular e Engenharia Genética/CBMEG, Faculdade de Ciências Médicas, Universidade Estadual de Campinas, 13083-970 Campinas, SP, Brazil; ^5^Departamento de Genética Médica, Faculdade de Ciências Médicas, Universidade Estadual de Campinas, Rua Tessalia Vieira de Camargo 126, 13083-970 Campinas, SP, Brazil; ^6^Departamento de Pediatria, Faculdade de Ciências Médicas, Universidade Estadual de Campinas, Rua Tessalia Vieira de Camargo 126, 13083-970 Campinas, SP, Brazil

## Abstract

Despite the increasing understanding of female reproduction, the molecular diagnosis of primary ovarian insufficiency (POI) is seldom obtained. The RNA-binding protein NANOS3 poses as an interesting candidate gene for POI since members of the Nanos family have an evolutionarily conserved function in germ cell development and maintenance by repressing apoptosis. We performed mutational analysis of *NANOS3* in a cohort of 85 Brazilian women with familial or isolated POI, presenting with primary or secondary amenorrhea, and in ethnically-matched control women. A homozygous p.Glu120Lys mutation in *NANOS3* was identified in two sisters with primary amenorrhea. The substituted amino acid is located within the second C2HC motif in the conserved zinc finger domain of NANOS3 and *in silico* molecular modelling suggests destabilization of protein-RNA interaction. *In vitro* analyses of apoptosis through flow cytometry and confocal microscopy show that NANOS3 capacity to prevent apoptosis was impaired by this mutation. The identification of an inactivating missense mutation in *NANOS3* suggests a mechanism for POI involving increased primordial germ cells (PGCs) apoptosis during embryonic cell migration and highlights the importance of NANOS proteins in human ovarian biology.

## 1. Introduction

Primary ovarian insufficiency (POI) is characterized by ovarian failure in women under the age of 40 years [1, 2]. POI may present as primary amenorrhea (PA) in severe cases with prepubertal onset or postpubertally as secondary amenorrhea (SA) associated with infertility, hypoestrogenism, and elevated gonadotropins (FSH > 40 U/L). This complex spectrum of progressive ovarian abnormality is largely related to the size of primordial germ cells (PGCs) pool, where prepubertal onset might reflect a complete lack of germ cells since birth causing a failure in the maintenance of ovarian somatic structure and postpubertal onset would reflect a variably insufficient pool of oocytes. This disorder is associated with female infertility and affects 1 to 2% of all women [3–5].

Several genetic mechanisms may lead to POI, including chromosomal abnormalities of the X chromosome or autosomes and autoimmune disorders [6]. Despite the increasing understanding of female reproduction, defined causes or mechanisms resulting in primary ovarian insufficiency remain largely unknown [7]. Persani et al. have estimated the prevalence of known genetic alterations in POI patients originally classified as idiopathic to be around 20 to 25% of cases [8], whereas others have found this prevalence to be lower, around 10%. Rare mutations have been described in genes involved in ovarian development and/or function such as* FSHR* (MIM 136435),* LHCGR* (MIM 152790),* BMP15* (MIM 300247),* POF1B* (MIM 300603),* NOBOX* (MIM 610934),* INHA* (MIM 147380),* GDF9* (MIM 601918),* NR5A1* (MIM 184757), and* FIGLA* (MIM 608697) and in meiotic genes [9–23]. Nevertheless, mutations in these genes account for a minority of cases of ovarian dysfunction, indicating that additional factors remain to be identified.

*Nanos* was first identified in* Drosophila*, where it represses the translation of target mRNAs through binding to their 3′ UTR and has a conserved function in germ cell development across species. Members of the evolutionarily conserved* Nanos* gene family are preferentially expressed in the ovaries and are known to play an important role in germ cell development, maintenance, and survival [24–30]. In Drosophila, the single* Nanos* gene (Nos) is required for development of the abdomen as well as for germ line maintenance [31, 32]. Three Nanos homologues exist in mouse, with Nanos2 and Nanos3 functioning primarily in male germ cell development and maintaining PGCs viability, respectively [33, 34]. In mice,* Nanos3* is expressed in the primordial germ cells (PGCs) from their formation until shortly after their appearance in the gonads (E13.5 in female and E14.5 in male embryos) [24]. Male and female mice deficient in* Nanos3* are infertile, and female *Nanos*3^−/−^ mice have atrophic ovaries in which no germ cells are detectable due to loss of migrating PGCs during embryogenesis [24]. PGCs are lost by apoptosis in the absence of* Nanos3*, establishing an essential function of* Nanos3* as a repressor of apoptosis in the germ cell [34].

A similar conserved function for NANOS proteins has been shown in humans. NANOS1 and NANOS2 seem to be mainly involved with male germ cell development and maintenance [35], and indeed NANOS1 mutations have been associated with male infertility manifested as nonobstructive azoospermia or oligozoospermia [36]. NANOS3, on the other hand, is expressed in both male and female fetal and adult gonads, and* in vitro* evidence suggests a pivotal function in human germ cell development [37].

Despite this compelling evidence for the importance of NANOS3 in germ cell maintenance, initial efforts to identify* NANOS3* mutations in women with POI were not successful: in 2007, Qin et al. analyzed 168 infertile Caucasian and Chinese women and failed to identify pathogenic mutations [38]. More recently, however, Wu et al. performed mutational analysis of the coding regions of* NANOS1*,* NANOS2*, and* NANOS3* in 100 Chinese POI patients, identifying a heterozygous p.Arg153Trp* NANOS3* mutation in a 23-year-old woman [39]. The mutant NANOS3 protein was shown to have decreased stability* in vitro*, leading the authors to postulate that reduced expression of NANOS3 in the ovaries would result in decreased PGC population and POI [39]. Herein we report a homozygous missense mutation in Nanos3 found in two sisters from our cohort of 85 Brazilian women with POI and present supporting* in vitro* functional data.

## 2. Materials and Methods

### 2.1. Subjects

This study was approved by the ethics committee of Hospital das Clinicas, University of Sao Paulo School of Medicine, Brazil (Protocol number 1226/07).

Eighty-five patients with POI were referred to the Developmental Endocrinology Unit of Hospital das Clinicas, Sao Paulo, or to the Pediatric Department of University of Campinas, in Sao Paulo, Brazil, for evaluation of primary amenorrhea (45 patients, 10 familial cases) or secondary amenorrhea (40 unrelated patients). The age at diagnosis of amenorrhea was 29.2 ± 5.9 years (18 to 39 yrs). Parental consanguinity was present in 80% of familial cases, and no other relevant familial history was reported. Each family had 2 affected individuals, and age at diagnosis of familial cases was 19.4 ± 6.2 years (14 to 36 yrs). FSH levels were elevated in all patients, ranging from 27 to 150 U/L in patients with primary amenorrhea and from 32 to 158 U/L in patients with secondary amenorrhea. Mutations in* FSHR*,* NR5A1*,* BMP15*, and* GDF9*, premutations in* FMR1*, and thyroid, adrenal, or ovarian autoimmune disorders had been excluded in all patients. Ethnically and age-matched (23 to 39  years old) women with normal menarche and menstrual cycles were invited to participate as controls. Institutional review board approval and written informed consent were obtained from all subjects before sample collection for DNA analysis.

### 2.2. Mutational Analysis of* NANOS3*

Two protein-coding transcripts of Nanos3 are described in the Ensembl database: a 581 bp transcript (ENST00000339133) resulting in a 192-residue protein and a 776 bp transcript (ENST00000397555) resulting in a 173-residue protein; only transcript ENST00000339133 is part of the human consensus coding sequence set (CCDS).

Following genomic DNA extraction from peripheral blood leukocytes, the entire exonic region and at least 15 bp of exon/intron boundaries of transcript ENST00000339133 (RefSeq NM_001098622.2) were PCR-amplified and submitted to direct automated sequencing in an ABI PRISM 310 (Applied Biosystems, Foster City, CA).

### 2.3. Apoptosis Detection by Flow Cytometry

A pCMV6-AC-GFP-NANOS3 expression vector (OriGene Technologies, Rockville, MD) was used as a template to generate a c.358G>A mutant using the QuikChange site-directed mutagenesis kit (Stratagene, La Jolla, CA) and specific primers (F: 5′ GGC GCC ACA CGT AAG CGC GCC CAC AC 3′; R: 5′ GTG TGG GCG CGC TTA CGT GTG GCG CC 3′). African green monkey kidney COS-1 cells were transiently transfected using Lipofectamine 2000 (Invitrogen, Carlsbad, CA) and analyzed 48 hours later with or without treatment with 5 mM sodium butyrate (Calbiochem, San Diego, CA). Cells were stained with Annexin-V conjugates (Molecular Probes Invitrogen, Carlsbad, CA) according to manufacturer's protocol. GFP and Annexin-V fluorescence were excited at 488 nm and 650 nm and emission measured using 530/30 nm and 660/30 band pass filters, respectively.

### 2.4. Confocal Microscopy

Confocal microscopy micrographs were obtained using a 488 nm laser line and light emitted between 500 and 600 nm for GFP detection and a 650 nm laser line and light emitted between 660 and 700 nm for red Annexin-V detection. Images were collected on a META LSM 510 laser scanning confocal microscope equipped with a 63.0 × 1.2 W objective (Carl Zeiss, Jena, Germany). The same settings for image acquisition and processing were applied for all samples to allow comparison of the fluorescence intensities among different samples.

### 2.5. Molecular Modeling of NANOS3

A model for mutated human NANOS3 was built using the resolved structure of L3MBTL1 (PDB-ID 2RI7) as template since NANOS3 structure has not yet been resolved. In order to find the best model for NANOS3, a Blast alignment with proteins available in PDB was performed. Highest homology was found with the crystal structure of Nanos from zebrafish (PDB-ID 3ALR) with 58% identity. However, this structure did not include the region where the p.Glu120Lys mutation is located. Therefore, we looked for another protein bearing similarities with NANOS3. Such protein was L3MBTL1. Its X-ray resolved structure presented the second highest homology with NANOS3 (33% of identity) and included the p.Glu120Lys mutant region. Molecular modeling was performed with the SwissModel web-served program and images examined and edited using the web-based BlueStar STING (CNPTIA-Embrapa, Brazil) and PyMOL softwares (freely available at http://www.pymol.org).

## 3. Results

### 3.1. Identification of NANOS3 Mutation

**Figure 1:**
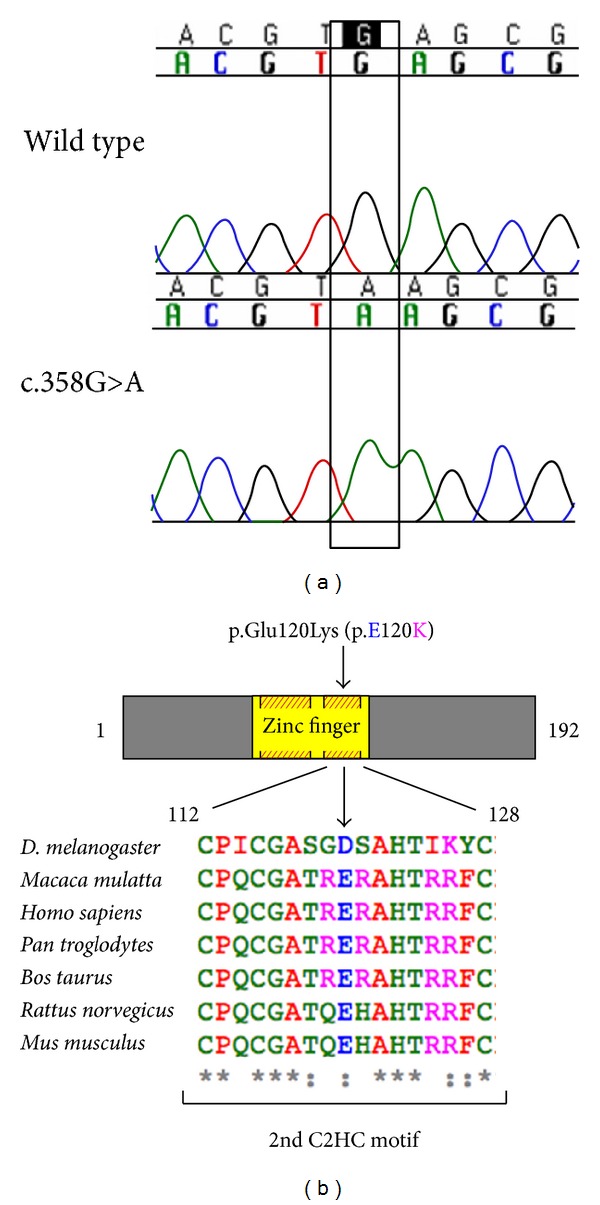
A Mutational analysis of* NANOS3* revealed a homozygous c.358G>A substitution in exon 1 in two sisters with primary amenorrhea. The GAG to AAG change in codon 120 results in a substitution from glutamic acid to lysine in the codified protein (p.Glu120Lys). B, Cartoon representation of the NANOS3 protein (192 amino acids), with the zinc finger domain (amino acid 76 to 130) depicted in yellow, showing the location of the p.Glu120Lys mutation. Glutamic acid 120 (shown as E120) is localized within one of two highly conserved Cys-Cys-His-Cys (C2HC) motifs (shaded red and yellow boxes). Conservation among species of the second C2HC motif sequence (human residues 112 to 128) is shown in detail; glutamic acid 120 (E) is highly conserved. Alignment was performed in Clustal Omega using protein sequences obtained from UniProt.

Mutational analysis of* NANOS3* revealed that two sisters carried a homozygous c.358G>A, p.Glu120Lys mutation (Figure 1), which was not detected in 113 ethnically matched control women and has not been reported in the 1000 Genome Project or dbSNP databases. Their mother was heterozygous and the father's DNA was not available for study. In the remaining 83 women composing our POI cohort, no new variants were identified in* NANOS3*. Two previously described SNPs were identified in our cohort, rs897790 and rs2016163 (Table 1).

The patients carrying the homozygous c.358G>A mutation were from the northeast region of Brazil (Piauí State) and born to nonconsanguineous parents. At 12 and 16 years old they were evaluated for breast underdevelopment. Both girls had not reached menarche, and physical examination revealed Tanner stage I breast development and Tanner stage III pubic hair. Basal gonadotropin levels were elevated with predominantly high FSH levels (*LH*⁡ = 63 and 21 U/L, FSH = 150 and 61 U/L, resp.). Estradiol levels were within the prepubertal range in both girls. Measured heights were 145 and 156 cm, and weights were 38 and 46 Kg, respectively. Pelvic ultrasonography showed no masses, and ovaries were not visualized. Treatment with conjugated estrogens followed by progesterone replacement resulted in complete breast development and normal cycling.

Their heterozygous mother has a history of menarche at 16 years old and difficulties to conceive. Her first pregnancy was at 27 years old after 4 years of attempts to conceive and her second and last gestation occurred at 30. Menopause occurred at 51 years of age.

### 3.2. Increased Apoptosis* In Vitro* with p.Glu120Lys Mutant NANOS3

**Figure 2:**
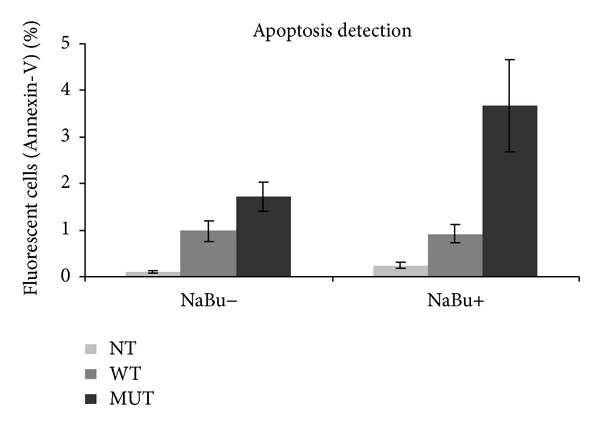
Detection of apoptosis using flow cytometry and Annexin-V staining in COS-1 cells transfected with wild type (WT) or p.Glu120Lys mutant (MUT) NANOS3-GFP, before and after induction of apoptosis with sodium butyrate (NaBu). Expression of mutant NANOS3 results in significantly higher apoptosis in comparison to wild type (*P* < 0.05). Error bars represent SEM for nine replicates; NT: nontransfected control.

Considering that Nanos3 has been shown to maintain germ cell development in model organisms through repression of apoptosis [34],* in vitro* studies were performed to assess the effects of the p.Glu120Lys substitution on the apoptotic rate of cultured cells. COS-1 cells were transfected with GFP-tagged wild-type or mutant NANOS3 and viability assessed by Annexin-V staining. Flow cytometry analysis showed that the percentage of Annexin-V stained GFP-positive cells was significantly higher in cells transfected with mutant NANOS3 in comparison to wild type, in untreated or sodium butyrate-treated cells (*P* < 0.05) (Figure 2), indicating that the p.Glu120Lys substitution profoundly impairs cell viability.

**Figure 3:**
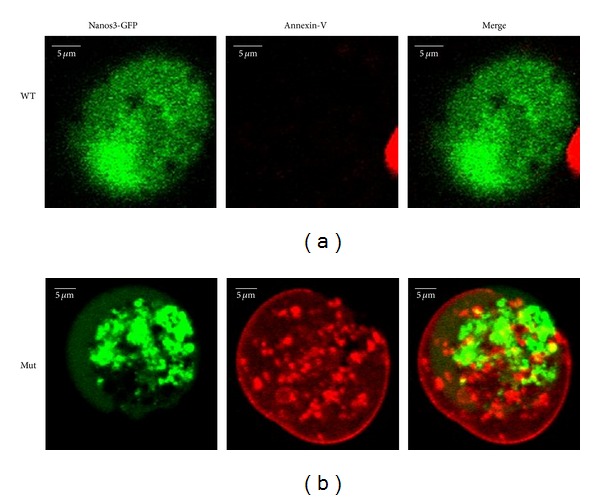
Confocal microscopy analysis of apoptosis in COS-1 cells following 48 h induction of apoptosis with sodium butyrate. In cells transfected with wild type (WT, upper row) or p.Glu120Lys mutant (Mut, lower row) GFP-tagged NANOS3, expression of green NANOS3-GFP is seen predominantly in the nucleus. Red Annexin-V staining in the cytoplasm and plasma membrane, indicating apoptosis, is seen in cells expressing p.Glu120Lys mutant NANOS3 but not in those expressing WT NANOS3.

Since NANOS3 has been shown to act in the cell nucleus [37], studies were performed to investigate whether the p.Glu120Lys mutation would affect intracellular localization. Transfected COS-1 cells were analyzed by confocal laser scanning microscopy for cellular localization of GFP-tagged wild type or mutant NANOS3 and Annexin-V staining (Figure 3). Nuclear localization was observed in cells transfected with both wild type and mutant cDNA. However, Annexin-V staining in the plasma membrane, indicating apoptosis, was only detected in cells expressing the p.Glu120Lys mutant NANOS3.

### 3.3. *In Silico* Mutant NANOS3 Modelling

**Figure 4:**
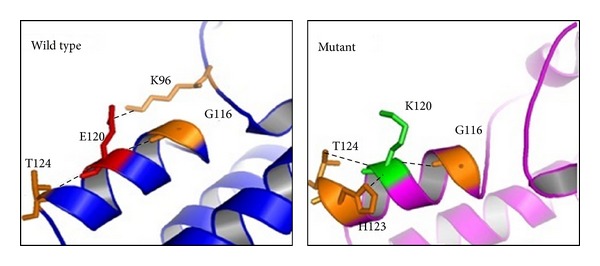
*In silico* analysis of amino acid interaction modification shows the different conformation conferred by the p.Glu120Lys substitution. Glutamic acid 120 (shown as E120) makes a hydrophobic interaction with lysine 96 (K96) and hydrogen bonds with both glycine 116 (G116) and threonine 124 (T124). The substitution to lysine 120 (K120) modifies structural points of contact establishing a new interaction with histidine 123 (His123) through aromatic stacking and abolishes the hydrophobic interaction with K96.

**Figure 5:**
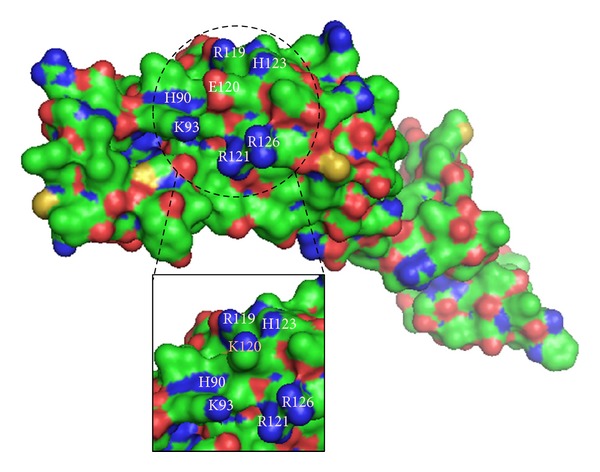
Bulk representation of wild-type NANOS3, with p.Glu120Lys mutant shown in details box. The dashed circle highlights a protein surface region rich in basic residues, shown in blue. At the center of this region, lies the acidic residue glutamic acid 120 (E120), shown in red. In the details, substitution by lysine 120 (K120) disturbs the electrostatic interactions among adjacent residues.

Glutamic acid120 is a negatively charged amino acid located within the zinc finger domain. This residue is found at the surface of NANOS3 within the second Cys-Cys-His-Cys (C2HC) motif, which is important for its ability to bind RNA [40]. In general, substitution of amino acids that share similar hydrogen binding capabilities and tridimensional requirements such as glutamic acid and lysine has limited structural consequences. However, lysine has a positive charge on the aliphatic side chain that can affect DNA binding activity when inserted into the zinc finger domain.* In silico* analysis shows that lysine 120 establishes a new contact with Lys96 and abolishes the original contact with His123 (Figure 4). Consequently, these changes could lead to internal contact conformational modifications resulting in impaired protein function. In addition, 3D structure indicates that the negatively charged glutamic acid 120 residue is surrounded by negatively charged residues (Figure 5). Therefore, the presence of the positively charged lysine 120 residue in the protein surface might cause strong electrostatic repulsion among the side chains and lead to destabilization upon protein-RNA interaction.

## 4. Discussion

The present study aimed to investigate the contribution of variants in NANOS3 to human POI. We have identified a nonsynonymous homozygous* NANOS3* mutation in two sisters with primary amenorrhea.

During development, once germ cells populate the gonads, they are exposed to several apoptotic waves. NANOS3 plays an important role in the maintenance and survival of primordial germ cells and therefore constitutes an interesting candidate gene for nonsyndromic POI. Indeed, NANOS3 has been shown to function in germ cell development in a breadth of organisms, from flies and worms through frogs and mammals. In mice, loss of* Nanos3* results in infertility due to apoptosis of migrating PGCs during fetal life, resulting in atrophic gonads where germ cells are absent [24, 34]. Furthermore, the contribution of* Nanos3* to PGC maintenance and survival seems to be dosage-dependent in mice, as a model with attenuated Nanos3 transcript levels displayed markedly reduced numbers of PGCs [39].

In humans,* NANOS3* is expressed in fetal and adult ovaries during multiple stages of oogenesis and* in vitro* studies of human embryonic stem cell-derived germ cells have shown coexpression of NANOS3 in nuclei with important germ cell factors such as BLIMP1, VASA, and STELLA in the cytoplasm [37]. In these cells, reduction of NANOS3 expression resulted in altered gene expression patterns and reduced number of cells in active division, suggesting that in humans too NANOS3 has an important function in germ cell maintenance and survival. Recently, a heterozygous p.Arg153Trp* NANOS3* mutation was reported in a 23-year-old Chinese woman out of a cohort of 100 women with POI [39]. Familial segregation was not defined, but the mutation was not found in 200 ethnically-matched controls.* In vitro* studies suggested decreased protein stability due to the p.Arg153>Trp mutation, leading the authors to postulate a mechanism of reduced dosage of NANOS3 expression in the ovaries leading to decreased PGC population and resulting in POI [39].

The p.Glu120Lys mutation identified in our patients lies within the NANOS3 zinc finger domain, which is composed of two C2HC motifs located between amino acids 77 and 131. The differences in chemical properties between amino acids might lead to impaired binding of NANOS3 to its mRNA targets, as suggested by protein modeling.* In vitro* experiments show that the mutant NANOS3 identified in the two sisters with POI was associated with increased apoptosis in transfected cells, suggesting that loss of NANOS3-mediated protective effect against apoptosis in primordial germ cells may be the mechanism underlying POI in these patients.

Primordial germ cell pool size in the developing ovary and maintenance of these cells throughout life are at the center of the pathogenesis of POI. It is remarkable that our two patients with homozygous* NANOS3* mutation have presented with primary amenorrhea, perhaps indicating more severely PGC-depleted ovaries, while their heterozygous mother had difficulty to conceive, which could represent a manifestation of insufficient PGC pool size. Indeed, the patient described by Wu et al. carried a heterozygous missense change and presented with secondary, rather than primary, amenorrhea [39], supporting the concept of a dosage effect for NANOS3-related POI.

In conclusion, we report a rare homozygous missense mutation in* NANOS3* in two Brazilian sisters with primary amenorrhea from a cohort of 85 Brazilian women with POI.* In vitro* and* in silico* functional studies support that this inactivating mutation abolishes NANOS3 ability to prevent apoptosis, suggesting a mechanism for POI involving increased PGC apoptosis during embryonic cell migration.

## Figures and Tables

**Table 1:** Polymorphic variants in *NANOS3* identified in POI patients and controls.

		Genotype frequency (%)					
Variant	dbSNP id	Patients with POI			Controls		
		Wild-type	Heterozygote	Homozygote	Wild type	Heterozygote	Homozygote
c.-23C>T (5′ UTR)	rs897790	60	34.1	5.9	72.5	25.7	1.8
c.353A>G (exon 1, synonymous)	rs2016163	50.6	40	9.4	70	27.4	2.6
